# Personality Traits and Family SES Moderate the Relationship between Media Multitasking and Reasoning Performance

**DOI:** 10.3390/jintelligence12060058

**Published:** 2024-06-03

**Authors:** Yuning Ma, Jinrong Yin, Hongzhou Xuan, Xuezhu Ren, Jie He, Tengfei Wang

**Affiliations:** 1 Department of Psychology and Behavioral Sciences, Zhejiang University, Hangzhou 310058, China; mayuning@zju.edu.cn (Y.M.); chtwyin@zju.edu.cn (J.Y.); hz.xuan@zju.edu.cn (H.X.); 2 School of Education, Huazhong University of Science & Technology, Wuhan 430074, China

**Keywords:** media multitasking, reasoning, personality traits, Family SES, moderation effect

## Abstract

The prevalence of media multitasking has raised concerns regarding its potential impact on cognitive abilities. Despite increasing attention given to this topic, there remains no consensus on how media multitasking is related to cognitive performance. This study aims to shed light on this issue by examining whether and how personality traits and family socioeconomic status (SES) moderate the relationship between media multitasking and reasoning performance. To this end, a large sample of university students (*n* = 777) completed a battery of measures, including the Raven’s Advanced Progressive Matrices, the Media Multitasking Inventory, the Big Five Inventory, the Barratt Impulsiveness Scale, the Grit Scale, and the Family SES Questionnaire. Results revealed a negative correlation between media multitasking and reasoning performance. However, this relationship was substantially moderated by conscientiousness, extraversion, openness, and family SES. Specifically, media multitasking was more detrimental to reasoning performance among individuals with lower levels of conscientiousness, extraversion, openness, and family SES, whereas it was less detrimental to counterparts with higher levels of these personality traits and family SES. The proposed moderation model, for the first time, not only offers novel insights into the theoretical accounts regarding how media multitasking relates to cognitive abilities, but also identifies the protective factors that may buffer the negative impacts of media multitasking.

## 1. Introduction

The widespread use of the Internet has led to increased accessibility of media devices and facilitated the prevalence of media multitasking. Media multitasking refers to the behavior of simultaneously using or switching between multiple types of media (e.g., using mobile social media while watching television) (Minear et al. 2013; Ophir et al. 2009). According to Nielsen (2018), adults typically engage with media for over 11 h daily, encompassing activities such as listening, watching, and reading. Multitasking accounts for approximately 96% of this duration (Deloitte 2019; Fisher et al. 2023). However, long-term engagement in media multitasking has been suggested to be negatively related to cognitive abilities, such as reasoning, executive functions, and working memory (Van der Schuur et al. 2015). Despite the growing number of studies examining these relations, theoretical accounts and empirical findings remain largely controversial. The present study therefore aims to shed new light on how media multitasking is related to reasoning performance by testing whether the relationship varies according to personal traits or family contexts.

### 1.1. Media Multitasking and Cognitive Abilities

There is an increasing number of studies exploring the relationship between media multitasking and cognitive abilities (e.g., Müller et al. 2021; Ophir et al. 2009; Uncapher and Wagner 2018; Van der Schuur et al. 2015). However, it remains ambiguous how media multitasking relates to cognitive abilities. Two contrasting hypotheses have been proposed to account for the relationship between the frequency of media multitasking and cognitive abilities (e.g., Ophir et al. 2009; Van der Schuur et al. 2015).

The scattered attention hypothesis posits that constant exposure to several media activities simultaneously is likely to get people into the ‘breadth-biased’ attention allocation pattern (i.e., pay attention to several tasks simultaneously rather than just focus on one task) (Ophir et al. 2009; Van der Schuur et al. 2015). People may become more easily distracted from their primary task when they frequently attend to large amounts of information. This presents a real challenge for people to filter irrelevant information from the environment given that they mostly attend to both relevant and irrelevant information (Ophir et al. 2009; Van der Schuur et al. 2015), which is likely to impair their cognitive functioning in the long run. According to the scattered attention hypothesis, people who engage in media multitasking more frequently possess lower levels of cognitive abilities than those who less often engage in media multitasking (e.g., Müller et al. 2021; Ophir et al. 2009). Consistent with this hypothesis, research shows that heavy multitaskers (HMMs) exhibited worse performance on tasks of executive functions, including updating (Luo et al. 2022), inhibition (Baumgartner et al. 2014; Murphy and Creux 2021), and shifting (Ophir et al. 2009) in comparison to light media multitaskers (LMMs). In a seminal study, Ophir et al. (2009) grouped participants to HMMs (1 SD or more above the mean) and LMMs (1 SD or more below the mean), and assessed their capacities of updating, shifting, and inhibition. Their results showed that HMMs were more likely to falsely respond to the previously presented stimuli than LMMs in the 3-back task but not in the 2-back task, suggesting that HMMs are more vulnerable to distractions as working memory loads increase. HMMs showed larger switch cost than LMMs, suggesting HMMs are less capable of flexibly switching between different task sets. The two groups did not differ in their performance on the stop-signal task. Except for executive functions, the frequency of media multitasking was also negatively associated with reasoning performance on the Raven’s Advanced Progressive Matrices (Minear et al. 2013) and the Logical Reasoning Test (Müller et al. 2021).

On the contrary, the trained attention hypothesis proposes that the behavior of media multitasking may serve as a training of cognitive functioning, because media multitaskers frequently engage in multiple activities simultaneously (e.g., Ophir et al. 2009). The frequent practice of cognitively demanding activities improves cognitive abilities (Cardoso-Leite et al. 2015; Van der Schuur et al. 2015). This means that people who frequently engage in media multitasking have better cognitive abilities than those who do not (Baumgartner et al. 2014; Murphy and Creux 2021). Consistent with this hypothesis, Murphy and Creux (2021) found that the frequency of media multitasking was positively associated with working memory capacity after controlling age, IQ, and attentional impulsiveness. Baumgartner et al. (2014) showed that frequent media multitaskers were better at resisting irrelevant distractions in the flanker task.

Apparently, there are contrasting hypotheses and evidence for the association between media multitasking and cognitive abilities. A complication to the situation is that a number of studies reported no relation between the frequency of media multitasking and cognitive abilities (Van der Schuur et al. 2015). Specifically, LMMs do not differ from HMMs in updating (Edwards and Shin 2017), inhibition (Luo et al. 2022; Müller et al. 2021; Ophir et al. 2009), shifting (Baumgartner et al. 2014; Luo et al. 2022; Minear et al. 2013; Müller et al. 2021), and reasoning (Loh and Lim 2020).

The mixed picture regarding the relationship between media multitasking and cognitive abilities has motivated us to clarify the factors or conditions that may affect the direction and magnitude of the relations (Himi et al. 2023; Van der Schuur et al. 2015). Since personality traits and family contexts are found to be associated with media multitasking behaviors (e.g., Chen et al. 2021; Nikken and Opree 2018; Toyama and Hayashi 2021; Wammes et al. 2019), it is conceivable that these factors may alter the consequences of media multitasking on cognitive performance. In the present study, we, for the first time, attempt to establish a moderation model to account for the inconsistency between different theories and empirical findings among university students.

### 1.2. Personality Traits as Potential Moderators for the Relationship

Personality reflects a stable and unique pattern of thinking, emotions, and behaviors (Ewen 2009). People with different personality traits usually show different patterns of behavior. Such a difference may account for the discrepancy of the relationship between media multitasking and cognitive abilities. We focused on a broad range of personality traits, not only including Big Five personality traits, but also impulsiveness and grit. These personality traits were selected because they have been shown to be closely linked to media multitasking behaviors (e.g., Toyama and Hayashi 2021; Wammes et al. 2019).

The Big Five personality model suggests there are five dimensions of personality including openness, conscientiousness, extraversion, agreeableness, and neuroticism (Costa and McCrae 1992). Conscientiousness reflects characteristics such as systematic, efficient, dutiful, and self-disciplined (Goldberg 1992). People with high conscientiousness typically complete tasks in a more systematic, efficient, and consistent way, and have better persistence and self-regulation in resisting distraction (Costa and McCrae 1992). As a consequence, they are less likely to engage in a secondary task when being distracted by irrelevant information, such as checking a pop-up message while reading an academic paper. This type of media multitasking behavior is assumed to be distractive media multitasking (Kraushaar and Novak 2010). Previous research shows that conscientiousness negatively predicted participants’ distractive media multitasking behavior, such as in-class phone use (Toyama and Hayashi 2021). Given that the scattered attention hypothesis presumes that media multitasking behavior due to being distracted by irrelevant information is associated with poor cognitive performance, it is thus assumed that the reasoning performance of people with lower conscientiousness would be more negatively associated with media multitasking than in those with higher conscientiousness.

Extraversion is characterized as being outgoing, sociable, energetic, and adventurous (Goldberg 1992). People of high extraversion are usually energetic, and prone to explore something unknown and new (John and Srivastava 1999). Typically, extraverts experience lower arousal than introverts when facing the same amount of stimulation because of their higher neurological threshold of arousal. This means that extraverts are more likely to simultaneously engage in multiple activities and are accustomed to higher levels of stimulation than introverts (Furnham and Allass 1999). The optimal level of arousal is usually associated with the best cognitive performance as suggested by activation theory (Gardner 1986). The arousal state incurred by media multitasking might be desirable to extraverts, but relatively high for introverts, resulting in negative impacts on introverts’ cognitive performance. Indeed, there is research showing that introverts were more likely to be disturbed by musical distraction (Furnham and Bradley 1997) or television distraction (Ylias and Heaven 2003) than extraverts during the reading comprehension and memory tasks. In another study, Furnham and Allass (1999) revealed that complex musical distractions even facilitated extraverts’ performance on the cognitive tasks, but impaired introverts’ performance. These findings suggest that media multitasking may be more negatively related to the reasoning performance of people with lower extraversion comparing to peers with higher extraversion.

Openness to experience reflects the characteristics of imagination, creativity, and being unconventional (Goldberg 1992). Individuals of higher openness are more willing to explore something unconventional, showing a “preference for variety” (McCrae and Costa 1997). According to this account, we speculate that people of higher openness may be more likely to actively engage in media multitasking to explore something new beyond the primary task, rather than being passively distracted by irrelevant information. Previous research demonstrated that people of higher openness reported fewer problematic media usages, such as problematic smartphone use (Horwood and Anglim 2018). Building on the scattered attention hypothesis, it is assumed that the reasoning performance of people with lower openness would be more negatively associated with media multitasking than those with higher openness. Regarding agreeableness and neuroticism, Toyama and Hayashi (2021) showed that agreeableness and neuroticism could not significantly predict the frequency of disruptive media multitasking behaviors (e.g., in-class phone use). Similarly, Wilson et al. (2010) revealed that agreeableness and neuroticism did not significantly predict addictive tendencies towards social networking sites. Based on these observations, it is conceivable that agreeableness and neuroticism might not affect media use habits and thus might not moderate the relationship between media multitasking and reasoning performance.

Grit is defined as perseverance and passion for long-term goals (Duckworth et al. 2007). People with higher grit usually work strenuously to overcome difficulties instead of giving up, maintain efforts and interests over years despite failures, and have better self-regulation so that they can continuously focus on their primary task without being distracted by irrelevant information (Duckworth et al. 2007). Research indicates that grit is negatively associated with mind wandering (Wammes et al. 2019), suggesting that people of higher grit are less likely to be distracted from the primary task (Forster and Lavie 2014). In addition, Parry and Roux (2019) found that grit negatively predicted individuals’ off-task media use in lectures, suggesting that individuals with lower grit may be more distracted by information unrelated to the primary task. This hints that media multitasking may be more negatively related to the reasoning performance of people with lower grit than peers with higher grit.

Impulsiveness refers to the personality trait characterized as responding quickly to a given stimulus, without deliberation and evaluation of consequence (Gerbing et al. 1987). People of higher impulsiveness usually act on a whim without deliberate planning and consideration of the consequences (Gerbing et al. 1987), exhibiting more digital distraction in the classroom (e.g., non-class related technology use, Chen et al. 2020). Accordingly, people of higher impulsiveness might be easily distracted by stimulation irrelevant to the primary task and engage in the secondary task. Therefore, media multitasking may be more negatively related to the reasoning performance of people with higher impulsiveness compared with counterparts with lower impulsiveness.

### 1.3. Family SES as a Potential Moderator for the Relationship

Different from personality traits, family SES is another crucial contextual factor that may moderate the relationship between media multitasking and cognitive performance. It refers to a family’s social and economic situation, which is usually indexed by family income, parents’ educational level, and parents’ occupations (Hollingshead 1975). We chose to explore the moderation role of family SES because low family SES may impede the development of attention abilities and the corresponding brain regions (Ursache and Noble 2016; Wray et al. 2017). For example, Wray et al. (2017) showed that participants from lower family SES families were associated with poor distractor suppression and were easily attracted by task-unrelated stimulation. This suggests that people from lower SES family may be more likely to engage in media multitasking due to their susceptibility to distraction, which could potentially harm cognitive performance.

In addition, family SES may influence the types of media multitasking one engages in (e.g., Kononova et al. 2019; Nikken and Opree 2018; Yang and Zhu 2016). For example, Kononova et al. (2019) revealed that individuals with higher family SES were more inclined to combine other activities with newspaper and magazine reading. By contrast, individuals with lower family SES were more prone to pair other activities with radio and television use. These two types of media multitasking are different. For higher family SES individuals, the secondary task requires active cognitive efforts and is more likely to be initiated by themselves. This type of multitasking is cognitively demanding and thus may be beneficial to them. For lower family SES counterparts, the secondary task is more likely caused by task-irrelevant stimulation since radio and television yield external sounds that easily grab one’s attention. Engaging in this type of multitasking may impair cognitive functioning according to the scattered attention hypothesis. Therefore, it is assumed that the reasoning performance of people with lower family SES would be more negatively associated with media multitasking than that of those with higher family SES.

### 1.4. The Present Study

To summarize, findings on the relationship between media multitasking and cognitive abilities have so far been mixed. The scattered attention hypothesis and trained attention hypothesis cannot account for the inconsistency. The inconsistency may be due to two primary reasons. First, previous studies mostly relied on small samples to examine media multitasking and its correlates, and even the extreme groups (i.e., HMMs and LMMs) were selected from a small sample, resulting in less reliable results. Second, to our knowledge, there is no study examining the moderators of the relationship. However, different people have quite diverse needs and habits when performing media multitasking, which, therefore, may lead to completely different consequences for them.

The current research aimed to explore whether personality traits and family SES modulated the relationship between media multitasking and reasoning performance. To this end, we collected data from a large sample among participants with diverse levels of media multitasking frequency, reasoning ability, personality traits, and family SES. We used the state-of-the-art Media Multitasking Inventory (Madore et al. 2020) to measure the frequency of media multitasking. We used Raven’s Advanced Progressive Matrices to measure individuals’ reasoning performance, for the reason that it has been recognized as a golden indicator of general fluid intelligence (e.g., Kvist and Gustafsson 2008; Peng et al. 2019) and is closely related to a wide range of cognitive abilities, such as executive functions (Friedman et al. 2006), working memory capacity (e.g., Unsworth et al. 2014; Wang et al. 2017), and attention (e.g., Ren et al. 2012). In addition, it has been used in previous media multitasking studies (Alzahabi et al. 2017; Minear et al. 2013) and is able to distinguish individual differences in reasoning performance. In addition, personality traits, impulsiveness, grit, and family SES were all assessed with well-established measures. Using both the correlation analysis and extreme group comparison, we examined the relationship between media multitasking and reasoning ability. More importantly, the moderation effects of Big Five personality traits, grit, impulsiveness, and family SES on the relationship between media multitasking and reasoning ability were tested. The findings of this study are expected to shed new light on the existing theories regarding how media multitasking relates to cognitive ability.

## 2. Materials and Methods

### 2.1. Participants

A total of 847 participants were recruited from a university located in Eastern China using a convenience sampling approach. The data of 67 participants were excluded from analyses since they did not complete all the measures. The data of another three participants were excluded for reporting impossible number of hours per week spent using specific forms of media (i.e., spending 150 h sending messages per week, spending 180 h listening to music per week, and spending 201 h watching videos per week). A final sample for analyses consisted of 777 participants (342 women; *M*_age_ = 19.11 years, *SD* = 1.07). We calculated the power of moderation analyses with G*Power 3.1.9.7 (Faul et al. 2007). A post hoc power analysis indicated that the effect of moderation analysis showed a high statistical power of 0.98, with a sample size of 777, and f^2^ = 0.02 (small effect size, Cohen 1988). This study was approved by the local research ethics committee. A written informed consent was obtained from all participants prior to the testing. University students were considered since they are allowed to use media devices more freely than primary and secondary school students in China.

### 2.2. Measures

#### 2.2.1. Media Multitasking

We used the Media Multitasking Inventory adapted by Madore et al. (2020) according to the original version (Ophir et al. 2009) to measure the frequency of media multitasking. The inventory was translated into Chinese. To ensure translation accuracy, the Chinese version was blindly back-translated into English, and then translated into Chinese again by a researcher proficient in both Chinese and English. The questionnaire consists of two sections: the Section 1 asks participants to report the number of hours per week they usually spend doing each of nine activities, including reading, homework (other than reading), watching videos, movies or TV, listening to music, radio, audiobooks or other audio, playing video games, browsing the Internet, texting or using social media or instant messaging, talking on the phone or video chatting, and other computer activities. The Section 2 requires participants to assess for each activity that how often they simultaneously engage in each of the other activities on a four-point Likert scale. Numeric values were assigned to participants’ estimations as follows: 1, most of the time; 0.67, some of the time; 0.33, a little of the time; and 0, never. Each media pairing was rated once, resulting in a total of 72 pairs. The Media Multitasking Index (MMI) was used to quantify individuals’ media multitasking frequency, and it was computed according to the Formulas (1) and (2) (Madore et al. 2020; Ophir et al. 2009):

(1) $$h_{total}=\sum_{i=1}^{9}h_{i}$$

(2) MMI=∑i=19(mi×hi)htotal

where *h_total_* is the total number of hours per week spent using all forms of media, m_i_ denotes participants’ estimations of the frequency of use on other media activities while using a primary medium *i*, and *h_i_* refers to the number of hours per week spent using the primary medium *i*.

#### 2.2.2. Reasoning Performance

Raven’s Advanced Progressive Matrices (APM, Raven et al. 1997) was employed to assess individuals’ reasoning ability. In this test, participants were asked to find the rules underlying a set of figures and use the rules to infer the missing one. Each item included a 3 × 3 matrix with the bottom right cell missing. Participants had to select a correct answer from the eight alternatives according to logical rules. Participants were given 10 min to complete the 18 odd-numbered items of the APM (similarly to Unsworth et al. 2014; Li et al. 2022). The items were displayed successively in an ascending order of difficulty. The score was the total number of correct responses, with a higher score indicating better reasoning performance. In the present study, the reliability coefficient (Cronbach’s alpha) of the APM was 0.61.

#### 2.2.3. Big Five Personality Traits

The Chinese version of Big Five Inventory-2 (BFI-2, Zhang et al. 2021) was used. This scale includes 60 statements, which are rated on a 5-point Likert scale from 1 (strongly disagree) to 5 (strongly agree). The scale consists of five dimensions, including neuroticism (e.g., Sometimes I feel angry and resentful), extraversion (e.g., I like to have lots of friends around me), openness (e.g., I like to cultivate and develop new hobbies), agreeableness (e.g., I try to be polite and very polite to everyone I meet), and conscientiousness (e.g., I will try my best to fulfill all the tasks assigned to me). Each of the five dimensions is assessed by 12 items. The score for each dimension is the mean score of the 12 items. A higher score in a specific dimension indicates that the individual aligns more closely with the description of that particular personality trait. For instance, if someone scores higher on the extraversion sub-scale, it suggests that they display more extraverted tendencies. The Chinese BFI2 exhibits good reliability, structural validity, convergent/discriminant validity, and criterion-related validity (Zhang et al. 2021). In the current study, all five dimensions of the scale (i.e., neuroticism, extraversion, openness, agreeableness, and conscientiousness) showed acceptable Cronbach’s alphas (i.e., 0.88, 0.85, 0.84, 0.82, and 0.86, respectively).

#### 2.2.4. Impulsiveness

The Chinese version of the Barratt Impulsiveness Scale (Li et al. 2011) consists of 30 items. This scale consists of three sub-scales, including Non-planning Impulsiveness (e.g., I plan tasks carefully), Motor Impulsiveness (e.g., I do things without thinking), and Cognitive Impulsiveness (e.g., I concentrate easily). Each sub-scale contains 10 items, and each item is rated on a five-point Likert scale (from “no” to “always”). The mean score of all items was computed, with a higher score indicating a higher extent of impulsiveness. The reliability and validity of the Chinese version of the Barratt Impulsiveness Scale are good (Li et al. 2011). In the present study, the reliability coefficient (Cronbach’s alpha) of this scale was 0.86.

#### 2.2.5. Grit

The Chinese version of the Original Grit Scale (Duckworth et al. 2007) was used to measure participants’ ability to persevere in difficult and even impossible tasks (Xiao et al. 2021). This scale consists of 12 items concerning persistence and interests for long-term goals (e.g., “I often set a goal but later choose to pursue a different one” and “I finish whatever I begin”). All items were rated on a 5-point Likert scale, from 1 (very much like me) to 5 (not like me at all). The mean score of all items was computed, with a higher score indicating a higher extent of self-perception of grit. The scale shows satisfactory reliability and validity (Xiao et al. 2021). In the present study, the reliability coefficient (Cronbach’s alpha) of this scale was 0.74.

#### 2.2.6. Family SES

A three-item questionnaire was used to measure family SES (Hollingshead 1975). One item assesses family income and two items assess parental educational level. Monthly family income is measured with a 6-point scale, with 1 = less than 1000, 2 = between 1001 and 3000, 3 = between 3001 and 6000, 4 = between 6001 and 10,000, 5 = between 10,001 and 30,000, and 6 = more than 30,001 CNY per month. The educational level of the parents was measured with a 6-point scale such that 1 = never went to school, 2 = primary school education, 3 = junior school education, 4 = senior school education, 5 = undergraduate education, and 6 = post-graduate education. The scores of each item were converted to z-scores. The family SES score was the average of three z-scores, with a higher score indicating a higher socioeconomic status. In the present study, the reliability coefficient (Cronbach’s alpha) of this questionnaire was 0.76.

### 2.3. Procedure

Participants were tested in groups in a quiet classroom with the supervision of a teacher. The measures were administered in the following order: Raven’s Advanced Progressive Matrices, the Media Multitasking Inventory, the Big Five Inventory, the Barratt Impulsiveness Scale, the Original Grit Scale, and the family SES Questionnaire. The testing took approximately 40 min.

### 2.4. Statistical Analysis

We identified outliers by examining between-subject distributions. Any observations exceeding 3 SDs from the means were defined as outliers, and subsequently replaced with a value that was M + 3 SDs for the scores exceeding 3 SDs above the mean and M − 3 SDs for the scores falling below 3 SDs from the mean (following Miyake et al. 2000). This procedure affected no more than 5‰ of observations. Correlation analyses and a *t*-test were conducted to investigate the relationship between media multitasking and reasoning performance. All statistical tests were two-tailed at a significance level of 0.05. As for the moderation analyses, we firstly calculated the z-score for each variable, and then performed multiple regression analyses to examine moderation effects.

## 3. Results

### 3.1. Descriptive Statistics

The descriptive statistics of all measures and the intercorrelations between the measures are shown in Table 1. The mean MMI score in the present sample was 2.17 (*SD* = 1.07), which was comparable to that (*M* = 2.71, *SD* = 1.19) reported by Madore et al. (2020). Cronbach’s alphas of all measures were larger than 0.60, suggesting acceptable reliability. The variables showed normal distributions with values of skewness and kurtosis smaller than two. Regarding the correlations among the variables (see Table 1), there was weak but significant correlation between MMI and reasoning performance (*r* = −0.08, *p* = .023), indicating that participants who multitasked more frequently performed worse on the reasoning test. In addition, MMI was significantly associated with conscientiousness (*r* = −0.14, *p* < .001), agreeableness (*r* = −0.09, *p* = .016), neuroticism (*r* = 0.18, *p* < .001), impulsiveness (*r* = 0.14, *p* < .001), and grit (*r* = −0.15, *p* < .001). MMI was not significantly related to extraversion, openness, and family SES.

### 3.2. Differences in Reasoning Performance between Heavy and Light Media Multitaskers

Participants who scored lower than the first quartile of the MMI were categorized as LMMs, and those higher than the third quartile as HMMs (following Minear et al. 2013). This procedure resulted in 194 light and 194 heavy media multitaskers. A *t*-test revealed a significant difference between HMMs (*M* = 10.87, *SD* = 2.31) and LMMs (*M* = 11.31, *SD* = 2.13) on reasoning performance, *t* = 1.97, *p* = .050, Cohen’s *d* = 0.20.

### 3.3. Moderating Roles of Personality Traits and Family SES in the Relationship between MMI and Reasoning Performance

First, the interaction terms of MMI with each personality trait and family SES were generated. In the regression model, the predictors including MMI, each personality trait or family SES, together with the interaction of MMI × personality trait or MMI × family SES were entered into the model. If the regression coefficient for the interaction term is significant, it suggests that the relation between MMI and reasoning performance is substantially moderated by personality trait or family SES. Table 2 presents the results for each moderation analysis.

**Figure 1:**
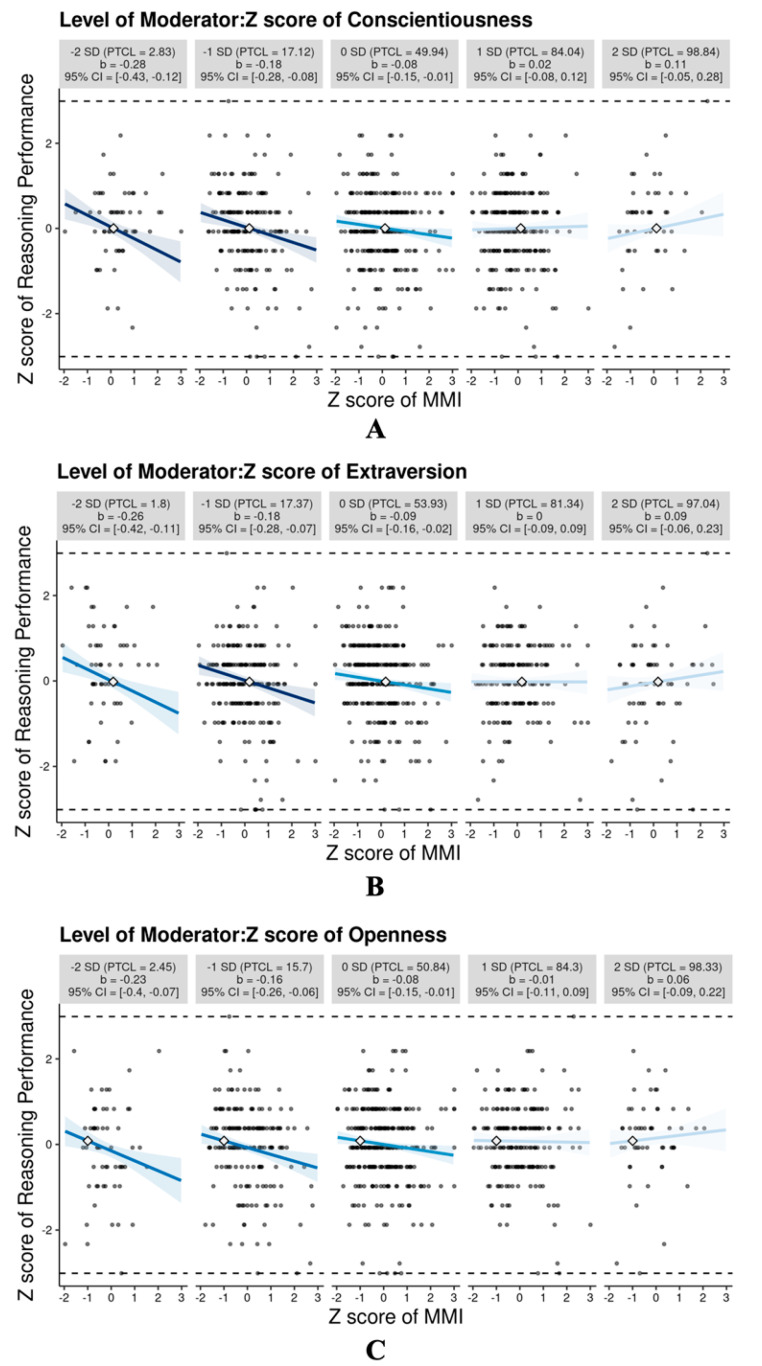
(**A**) Moderating role of conscientiousness in the relationship between media multitasking index (MMI) and reasoning performance. Simple slopes are presented for levels of the conscientiousness score 2 SD and 1 SD below the mean, at the mean, and 1 SD and 2 SD above the mean. Each graphic illustrates the computed 95% confidence region (shaded area), the observed data (gray circles), the maximum and minimum values of reasoning performance (dashed horizontal lines), and the crossover point (diamond). The x-axes denote the full range of MMI. CI: confidence interval; PTCL: percentile. The illustration was generated by means of the interActive software (McCabe et al. 2018). (**B**) Moderating role of extraversion in the relationship between media multitasking index (MMI) and reasoning performance. (**C**) Moderating role of openness in the relationship between media multitasking index (MMI) and reasoning performance.

Conscientiousness significantly moderated the relationship between MMI and reasoning performance (see Model 1). As shown in Figure 1A, there were significant negative correlations between MMI and reasoning performance when participants’ conscientiousness scores were lower than the average (in particular, *β* = −0.28 for people whose conscientiousness scores were 2 SDs below the mean). The magnitude of the correlation decreased when participants were more conscientious. It turned out to be insignificant when participants’ conscientiousness scores were higher than the average.

Extraversion significantly moderated the relationship between MMI and reasoning performance (see Model 2). As depicted in Figure 1B, MMI was negatively correlated with reasoning performance among the introverted participants whose extraversion scores were lower than the average (in particular, *β* = −0.26 for people whose extraversion scores were 2 SDs below the mean). The magnitude of the correlation decreased when participants were more extraverted. The correlation became insignificant among the participants whose extraversion scores were higher than the average.

Openness significantly moderated the relationship between MMI and reasoning performance (see Model 3). As depicted in Figure 1C, MMI was negatively associated with reasoning performance among participants whose openness scores were lower than the average (in particular, *β* = −0.23 for people whose openness scores were 2 SDs below the mean). The correlation decreased when participants were more open to experience. As the openness scores were higher than the average, media multitasking was not detrimental to reasoning performance. The moderating effects of impulsiveness, grit, agreeableness, and neuroticism on the relationship between MMI and reasoning performance were insignificant (see Models 4–7; Figures for these insignificant moderation effects are included in Appendix A).

**Figure 2:**
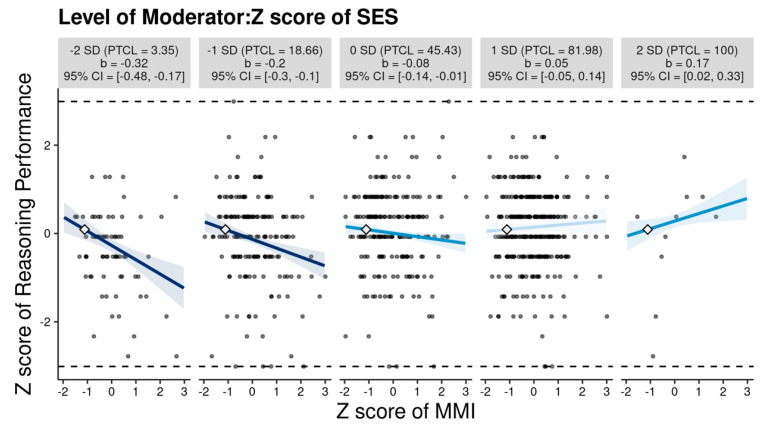
Moderating role of family socioeconomic status (SES) in the relationship between media multitasking index (MMI) and reasoning performance.

The moderating effect of family SES was significant (see Model 8). As shown in Figure 2, MMI was negatively correlated with reasoning performance among participants whose family SES was lower than the average (in particular, *β* = −0.32 for people whose family SES was 2 SDs below the mean). The correlation decreased as family SES increased. Surprisingly, for people whose family SES was 2 SDs above the mean, MMI turned out to be beneficial to reasoning performance (*β* = 0.17, 95% *CI* = 0.02–0.33).

## 4. Discussion

Media multitasking has undoubtedly become a daily habit nowadays. A number of studies have explored the relationship between media multitasking and cognitive abilities (Müller et al. 2021; Ophir et al. 2009; Van der Schuur et al. 2015), but existing results are mixed. Expanding upon the discrepancy in prior research, the present study, for the first time, examined whether personality traits and family SES modulated the relationship between media multitasking and reasoning performance. The results based on a large sample indicate that the frequency of media multitasking is negatively correlated with reasoning performance. Intriguingly, the relationship was substantially moderated by conscientiousness, extraversion, openness, and family SES.

Both the correlational analyses and extreme group comparison indicated that the frequency of media multitasking was negatively related to reasoning performance. This result is consistent with a few previous studies, indicating that LMMs outperform HMMs on the APM (Minear et al. 2013) and the other reasoning measures (e.g., LSP4, Müller et al. 2021). These findings corroborate the scattered attention hypothesis, suggesting that media multitasking negatively influences cognitive performance. However, there is also a study showing no relationship between media multitasking and reasoning performance (Loh and Lim 2020). The inconsistency may be because the relationship is affected by a third variable. Our study further sheds light on this issue by specifying the moderating roles of personality traits and family SES in the relationship. It should be noted that the correlation between MMI and reasoning performance was weak and should be explained cautiously. Nevertheless, there were typical and relatively large correlations (Gignac and Szodorai 2016) between MMI and reasoning performance (*β* = −0.23–−0.32) for people whose conscientiousness, extraversion, openness, or family SES was 2 SDs below the mean, suggesting the necessity to consider the moderators underlying the correlation.

Our results demonstrate that a few personality traits and family SES moderate the relationship between media multitasking and reasoning performance. To be specific, we found that media multitasking was more detrimental to reasoning performance when participants were less conscientious. This result suggests that conscientiousness serves as a protective factor that keeps people away from distractions when they are working on a primary task. That is, individuals with higher conscientiousness may engage in less distractive media multitasking behaviors, such as attending to information alerts from a mobile device while reading magazines. On the contrary, distractive media multitasking such as in-class phone use (Toyama and Hayashi 2021) may occur more often among low conscientiousness individuals, leading to impairment in cognitive functioning as the scattered attention hypothesis suggests.

Extraversion played a moderating role in the relation between media multitasking and reasoning performance. Among introverts (i.e., whose extraversion score was lower than the average), we observed negative correlations between media multitasking and reasoning performance. However, the correlation became insignificant among extraverts (i.e., whose extraversion score was higher than the average). Extraverts are usually energetic and tend to seek high levels of stimulation (John and Srivastava 1999). Thus, they are more accustomed to media multitasking that may lead to a desirable level of arousal (Furnham and Allass 1999). In contrast, the arousal level incurred by media multitasking may be high and even disruptive to introverts. This explains why media multitasking only showed detrimental effects on reasoning performance of introverts.

As for openness, higher frequency of media multitasking was related to worse reasoning performance only for participants who were less open to experience, but not for their high-openness counterparts. Individuals of high openness are more willing to explore something novel and unconventional, showing a “preference for variety” (McCrae and Costa 1997). Consequently, they are more likely to initiate a secondary task beyond the primary task, rather than being passively distracted by another irrelevant media task (Horwood and Anglim 2018). This explains why media multitasking did not exert negative impacts on the reasoning performance of individuals with higher openness.

In addition to personality traits, we found that family SES also modulated the relationship between media multitasking and reasoning performance. Two alternative accounts can explain this result. First, parents of high family SES families possess higher educational levels and may be better at instructing how to effectively use different media (Kononova et al. 2019). This suggests that people with higher family SES even benefit from media multitasking. However, due to the lack of guidance on the use of various media at home, people of low family SES may develop distractive media multitasking habits that eventually impair cognitive functioning. Second, lower family SES has been found to be associated with more attention problems (e.g., Ursache and Noble 2016), and higher susceptibility to task-unrelated distractions (Wray et al. 2017). People from low-family SES families are more likely to engage in media multitasking induced by irrelevant stimulation, resulting in the ‘‘breadth-biased’’ attention allocation pattern. This explains why media multitasking exerted negative influences on cognitive performance of individuals with lower family SES.

**Figure A1:**
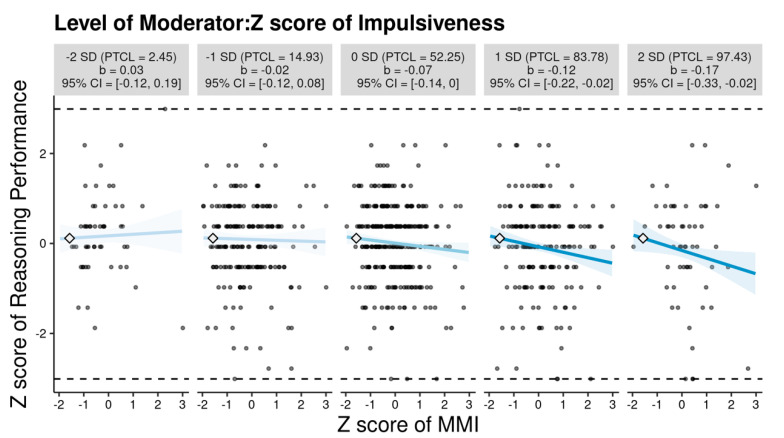
Moderating role of impulsiveness in the relationship between media multitasking index (MMI) and reasoning performance. Simple slopes are presented for levels of the conscientiousness score 2 SD and 1 SD below the mean, at the mean, and 1 SD and 2 SD above the mean. Each graphic illustrates the computed 95% confidence region (shaded area), the observed data (gray circles), the maximum and minimum values of reasoning performance (dashed horizontal lines), and the crossover point (diamond). The x-axes denote the full range of MMI. CI: confidence interval; PTCL: percentile. The illustration was generated by means of interActive software (McCabe et al. 2018).

**Figure A2:**
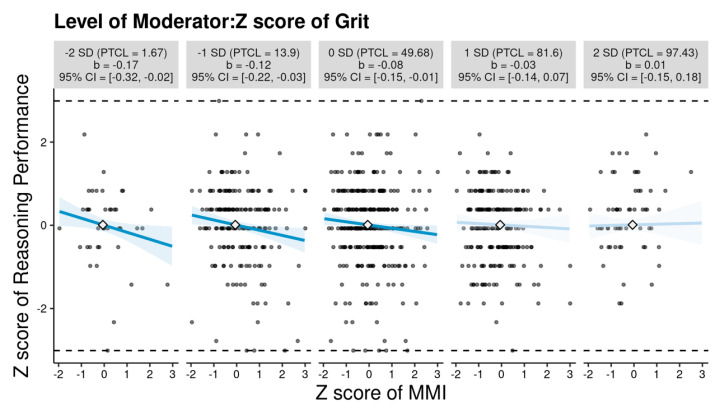
Moderating role of grit in the relationship between media multitasking index (MMI) and reasoning performance.

Although the moderating effects of impulsiveness or grit were insignificant, the trends for both moderating effects were also in accordance with our hypotheses. As is shown in Figure A1 and Figure A2 of Appendix A, there was significant negative correlation between MMI and reasoning performance when people scored higher than the mean score of impulsiveness or lower than the mean score of grit, whereas the correlations were insignificant for the other participants. The insignificance might be because the variance of these two variables was small in our sample, so the moderating effects did not reach the level of significance. To compare the variation of different variables, we computed coefficient of variation (CV = SD/Mean) of each variable, and found that CVs of grit (0.15) and impulsiveness (0.15) were smaller than the other significant moderators (ranging from 0.18 to 0.22). Therefore, it warrants more studies to investigate these moderation effects with a sample of higher variability in the moderators.

**Figure 3:**
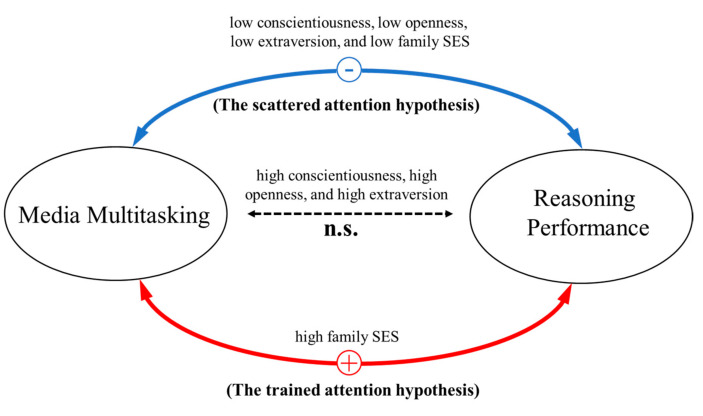
The moderation model of the relationship between media multitasking and reasoning performance. The blue line represents the significant negative correlations between media multitasking and reasoning performance, which is consistent with the scattered attention hypothesis. The red line represents the significant positive correlations between media multitasking and reasoning performance, which is consistent with the trained attention hypothesis. The dashed line denotes insignificant correlations.

Our findings shed new light on the theories regarding how media multitasking relates to reasoning performance. There are disparate theoretical accounts on the relationship. The scattered attention hypothesis regards media multitasking as disruptive behaviors, suggesting that people are distracted by secondary media tasks while completing the primary media task. However, the trained attention hypothesis assumes that media multitasking gives chances for people to train their multitasking ability and is thus beneficial to their cognitive performance. We propose a moderation theory to reconcile the discrepancy between the two hypotheses (see Figure 3). This theory outlines the factors that may influence how media multitasking is related to reasoning performance. That is, when people are less conscientious, less extraverted, less open to experiences, or lower in family SES, media multitasking is likely to be negatively associated with reasoning performance, which is consistent with the scattered attention hypothesis. When people are more conscientious, more extraverted, or more open to experiences, the relations between media multitasking and reasoning performance are insignificant. On the contrary, when people are from high-family SES families, more media multitasking may be related to higher reasoning performance, which is consistent with the trained attention hypothesis.

Revealing the moderating roles of personality traits and family SES in the relationship between media multitasking and reasoning performance is also enlightening for educators and parents with regard to media multitasking instruction. Our findings suggest the necessity of implementing targeted and individualized instructions on how to effectively use multiple media for people of different personality traits and family SES backgrounds. For example, for people of high conscientiousness, media multitasking does not have a negative effect on their cognitive ability. Therefore, they can be given more freedom to autonomously use media devices. For low-conscientiousness people, however, media multitasking has a detrimental effect on their cognitive ability. Educators and parents should create a quiet and distraction-free environment for them to focus on the primary task. Furthermore, our research underscores the importance of considering family SES in media multitasking instruction. For people from lower family-SES backgrounds, they may be more easily distracted by media multitasking due to undesirable media multitasking behaviors. It is advisable to provide additional instructions for them to develop constructive media multitasking behaviors.

In the digital age, attention is a scarce resource due to information overload and constant distractions. Recent research (Brasel and Gips 2017; Di Plinio et al. 2019) highlights how device intrusion may alter cognitive processes, particularly perception and sense of agency. The presence of media devices and constant notifications can impact cognitive control, attention spans, and decision-making abilities (Cardoso-Leite et al. 2015; Jung and Kowalski 2022). These findings emphasize the broader cognitive and behavioral impacts of media multitasking in contemporary digital environments, enriching our understanding of how individuals navigate and prioritize tasks amidst pervasive digital distractions. Adopting strategies like agenda setting, multitasking strategies, life/time management, and commonsense approaches to avoiding distractions and shortening attention spans becomes increasingly crucial in mitigating the detrimental effects, ultimately enhancing productivity and well-being (Jung and Kowalski 2022).

Several limitations of the current study and future directions should be noted. First, we merely examined the moderation models for the relationship between media multitasking and reasoning performance. Though reasoning is a representative indicator of individuals’ general cognitive ability, we still need to take caution when generalizing the conclusions to other cognitive abilities. Thus, future studies are suggested to examine the moderation model by including cognitive abilities, such as executive functions, memory, and attention. Second, we should be cautious to draw any causal relationship between media multitasking and cognitive abilities due to the cross-sectional nature of this study. Future research is recommended to collect data at multiple time points and investigate the cross-lagged relations between media multitasking and cognitive abilities. Thirdly, the present study merely included university students; however, different populations, such as adolescents, emerging adults, or elderly individuals, may exhibit varying levels of sensitivity and susceptibility to the effects of media multitasking on cognitive performance. In addition, adolescents and emerging adults may exhibit different levels of personality traits compared to older adults (Bleidorn et al. 2022). Therefore, future research should include a broader range of participants to further examine the moderation role of personality traits in the relationship between media multitasking and cognitive abilities. Finally, although we found that personality traits and SES can moderate the relationship between media multitasking and reasoning performance, it is still unclear what the underlying mechanism is. Future research can further compare differences in media multitasking behaviors among people with different personality traits to explain these moderating effects more clearly. For example, some researchers have proposed that there are productive and distractive media multitasking behaviors (Baumgartner and Wiradhany 2022; Kraushaar and Novak 2010), and these two types of media multitasking may exert different impacts on cognitive functioning. These research directions can further enhance our understanding of the complex relationship between media multitasking and cognitive abilities.

## 5. Conclusions

In summary, our study revealed a negative correlation between media multitasking and reasoning performance, with conscientiousness, extraversion, openness, and family SES significantly moderating this link. Specifically, lower levels of these traits and family SES were associated with greater detrimental effects of media multitasking on reasoning performance. These findings offer valuable insights into understanding the impact of media multitasking on cognitive abilities and highlight the importance of considering personal characteristics and socioeconomic context in exploring this relationship.

## Figures and Tables

**Table 1:** Descriptive statistics for the measures and the intercorrelations among them (*n* = 777).

	1	2	3	4	5	6	7	8	9	10
1. MMI	-									
2. APM	−0.08 *	-								
3. Extraversion	0.02	−0.02	-							
4. Agreeableness	−0.09 *	−0.05	0.21 ***	-						
5. Conscientiousness	−0.14 ***	−0.00	0.28 ***	0.42 ***	-					
6. Neuroticism	0.18 ***	−0.07	−0.33 ***	−0.47 ***	−0.39 ***	-				
7. Openness	0.00	0.07 *	0.38 ***	0.15 ***	0.27 ***	−0.11 **	-			
8. Impulsiveness	0.14 ***	−0.10 **	−0.21 ***	−0.34 ***	−0.67 ***	0.42 ***	−0.37 ***	-		
9. Grit	−0.15 ***	0.01	0.30 ***	0.35 ***	0.65 ***	−0.40 ***	0.28 ***	−0.62 ***	-	
10. Family SES	−0.05	0.16 ***	0.17 ***	−0.00	0.10 **	−0.06	0.25 ***	−0.20 ***	0.14 ***	-
Mean	2.17	11.16	3.01	3.72	3.43	2.87	3.59	2.10	6.48	0.00
SD	1.07	2.12	0.66	0.53	0.62	0.71	0.63	0.31	1.00	0.82
Skew	0.58	−0.49	0.14	−0.19	−0.20	0.23	−0.16	0.00	0.17	−0.57
Kurtosis	0.19	0.98	−0.45	0.03	−0.23	−0.19	−0.16	−0.05	0.02	−0.20
Reliability	−	0.61	0.85	0.82	0.86	0.88	0.84	0.86	0.74	0.76

Note. MMI: Media Multitasking Index; APM: Raven’s Advanced Progressive Matrices; SES: Socioeconomic Status. * *p* < .05, ** *p* < .01, *** *p* < .001.

**Table 2:** Multiple regression analyses predicting reasoning performance by MMI, personality traits measures, family SES, and their interactions (*n* = 777).

Predictor	*β*	*SE*	*t*	*p*
Model 1 (*F* = 4.12, *p* = .007, *R*^2^ = 0.02)				
MMI	−0.08	0.04	−2.25	0.025
Conscientiousness	−0.01	0.03	−0.30	0.764
MMI × Conscientiousness	0.10	0.04	2.65	0.008
Model 2 (*F* = 3.98, *p* = .008, *R*^2^ = 0.02)				
MMI	−0.09	0.04	−2.50	0.013
Extraversion	−0.02	0.03	−0.48	0.632
MMI × Extraversion	0.09	0.03	2.54	0.011
Model 3 (*F* = 4.39, *p* = .004, *R*^2^ = 0.02)				
MMI	−0.09	0.04	−2.39	0.017
Openness	0.08	0.03	2.18	0.030
MMI × Openness	0.07	0.04	1.99	0.047
Model 4 (*F* = 3.11, *p* = .026, *R*^2^ = 0.01)				
MMI	−0.09	0.04	−2.41	0.016
Agreeableness	−0.06	0.04	−1.61	0.107
MMI × Agreeableness	0.04	0.04	1.22	0.221
Model 5 (*F* = 3.31, *p* = .020, *R*^2^ = 0.01)				
MMI	−0.07	0.04	−1.99	0.047
Neuroticism	−0.06	0.04	−1.54	0.125
MMI × Neuroticism	−0.05	0.03	−1.52	0.129
Model 6 (*F* = 4.31, *p* = .005, *R*^2^ = 0.02)				
MMI	−0.07	0.04	−1.95	0.052
Impulsiveness	−0.09	0.03	−2.37	0.018
MMI × Impulsiveness	−0.05	0.04	−1.48	0.139
Model 7 (*F* = 2.27, *p* = .079, *R*^2^ = 0.01)				
MMI	−0.08	0.04	−2.15	0.032
Grit	0.00	0.04	0.09	0.929
MMI × Grit	0.05	0.04	1.28	0.203
Model 8 (*F* = 11.82, *p* < .001, *R*^2^ = 0.04)				
MMI	−0.08	0.04	−2.13	0.033
Family SES	0.15	0.04	4.23	<0.001
MMI × Family SES	0.11	0.04	3.18	0.002

Note. MMI: Media Multitasking Index; SES: Socioeconomic Status.

**Figure A3:**
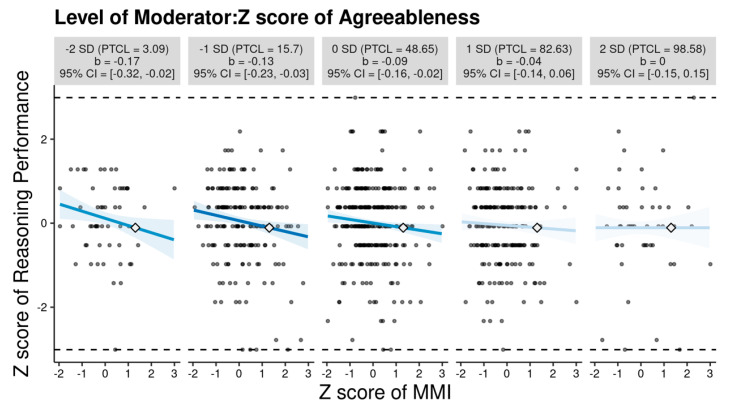
Moderating role of agreeableness in the relationship between media multitasking index (MMI) and reasoning performance.

**Figure A4:**
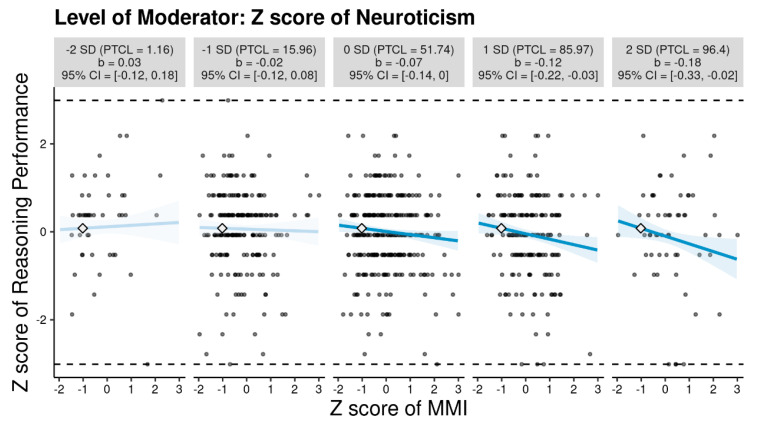
Moderating role of neuroticism in the relationship between media multitasking index (MMI) and reasoning performance.

## Data Availability

The data supporting the findings of this study and the syntaxes for data analyses are available at https://osf.io/6dwps/ (accessed on 6 May 2024).

## Appendix A

The Appendix A includes four figures, which are Figure A1, Figure A2, Figure A3 and Figure A4.
